# Enhancement of post-activation performance enhancement by blood flow restriction following specific on-ice exercise program in ice hockey players

**DOI:** 10.3389/fspor.2025.1659724

**Published:** 2025-09-12

**Authors:** Tomasz Gabrys, Radoslaw Chruscinski, Urszula Szmatlan-Gabrys, Michal Garnys, Marta Bichowska-Paweska, Ladislav Cepicka

**Affiliations:** ^1^ Sport Centrum Faculty of Education, University of West Bohemia, Pilsen, Czechia; ^2^ Department of Sport Science, 4Sport LAB, Warsaw, Poland; ^3^ Silesian Ice Hockey Federation, Katowice, Poland; ^4^ Faculty of Health Science, University of Applied Science, Nysa, Poland; ^5^ Faculty of Rehabilitation, Department of Anatomy, University of Physical Education, Krakow, Poland; ^6^ Department of Physical Education, Gdansk University of Physical Education and Sport, Gdansk, Poland

**Keywords:** post-activation performance enhancement (PAPE), blood flow restriction (BFR), vertical jump (CMJ, SJ), lower limb power, ice hockey performance, on-ice conditioning program

## Abstract

**Introduction:**

Due to the requirements for research on motor skills in elite hockey players, an attempt was made to assess the skills following the application of PAPE and BFR-enhanced PAPE to enhance lower limb power.

**Methods:**

An on-ice exercise program was used to determine PAPE factors that included three sets of 3 repetitions of specific on-ice effort, 15 s of work, 15 s of rest, separated by 90 s of free skating. The evaluation of the effectiveness of the PAPE program was measured at the 3rd, 6th, 9th, 12th, and 15th minutes after its application. The flight time during the CMJ and SJ jump was recorded with the OptoJump system, and the jump height (JH) and peak power (PP) were determined. The study involved 20 professional male hockey players aged 17 ± 1 years with 11 ± 2 years of training experience.

**Results:**

No differences between JH and PP values in CMJ and SJ before and after PAPE and PAPE with BFR intervention were statistically significant. The BFR application during specific effort showed a statistically significant *p* ≤ 0.001 increase in work time.

**Discussion:**

Significant individual differences in the magnitude of the PAPE and BFR effect between the subjects were also found. The individualization of exercise stimuli should consider the individual athlete's profile in terms of susceptibility to the PAPE and BFR program, considering the amount of fatigue it may cause.

## 1 Introduction

For the training effect, it is important to approximate the solutions to those characteristics of the starting exercises and the conditions in which the training is carried out. Warm-ups incorporate the above elements, preparing muscles for specific tasks or required intensity (1–4). At the same time, performing exercises with sub- and maximum power necessitates supporting the process through established methods combined with innovative sports training solutions. Post-activation potentiation (PAP) is a phenomenon well described in the literature that increases muscle force production with submaximal muscle activation (5). The application of PAP in training is difficult due to its dependence on electrostimulation (6) and the transient nature of its effect, which typically lasts up to 3 min (7), while peak force enhancement occurs within 5–10 min (8). Therefore, PAPE is a viable alternative, accounting for the differing time dynamics and mechanisms underlying strength augmentation (9). The distinction between PAP and Post-Activation Performance Enhancement (PAPE) primarily lies in their temporal characteristics (10). PAPE specifically refers to the performance improvement following high-intensity voluntary conditioning contractions, with empirical evidence demonstrating its efficacy in sport-specific warm-ups (11–14). The results of these studies confirm the functionally significant effect of PAPE in training practice. According to the latest research, the PAPE effect is enhanced by introducing blood flow restriction (BFR) at a specified time preceding exercises requiring high mobilization of muscle contraction (15).

The use of exercises with BFR to enhance the quality of strength and power training performed at maximum intensity has recently gained importance. This method of training support is used, among others, in team sports (16–19), including ice hockey, where high-power, short-duration efforts are crucial for creating goal-scoring opportunities. The combination of interval training with different intensities using BFR was studied by Lixandrão et al. and de Oliveira et al. (19, 20). However, despite numerous studies, there are no universal protocols for the use of BFR, and the effect of BFR on increasing the force of muscle contraction is also not unequivocal (21). For example, authors Driller and Overmayer (22) examined the impact of BFR on tissue oxygenation and muscle activation, where, during the study, participants performed a series of exercises under conditions of limited blood flow through the thigh muscle tissue. In the above studies, a reduction in tissue oxygenation was observed, but no significant increase in muscle activation was demonstrated. In turn, Huang et al. (23) demonstrated an immediate improvement in exercise performance and a sustained effect on physical fitness when BFR was applied to the ankle joint, highlighting its strategic application within a training unit prior to specific exercises. In another study, Behm et al. conducted a comparative analysis of the results of studies on the use of vascular occlusion training in the context of changes in flexibility, muscle size, and endurance. The authors found that BFR training can improve flexibility, muscle size, and endurance (24). However, it should be noted that no universal approach to the application of BFR has been established, and opinions regarding its effectiveness in power development remain divided. However, it should be remembered that the optimal pressure should be adjusted to the individual characteristics of each athlete, such as limb circumference, muscle strength, body fat, and cardiovascular fitness (25–27). However, the same value of AOP (e.g., 178 mmHg) can be used during the BFR exercise if all athletes have similar physical conditions and limb circumferences (27, 28). Moreover, the use of the same value for all participants has been described in previous studies (28) and reported as a reliable method for identifying sub-occlusive pressures (29). Therefore, if it is not possible to determine an individual AOP for each athlete due to, for example, time constraints during training, a standard pressure can be used (27).

There is no clear evidence of the influence of BFR on the power generated during jumping, as confirmed by the studies of Rodrigo-Mallorca et al. (30). However, using BFR bands has been shown to increase the range of motion (ROM) but does not affect the strength of the lower limb muscles. Also, Jianhong et al. (31) indicated, based on an analysis of 23 published studies, that the use of BFR can increase muscle strength, shorten the time of force development, and improve functional efficiency in exercises such as squats, sprints, and jumps (high power participation in the effective execution of the movement pattern). Further, previous studies have not evaluated the effectiveness of BFR in enhancing jump height, peak power and flight time during CMJ and SJ in professional hockey players, despite high-power output being crucial for their motor preparation (32, 33). Therefore, it is an important element of training and assessment of physical preparation in this sport. To fill this knowledge gap, research was conducted on BFR applied during sport-specific high-intensity on-ice effort would influence countermovement jump (CMJ) and squat jump (SJ) performance in professional ice hockey players, with potential differences in the magnitude and duration of these effects compared to standard conditioning.

## 2 Methods

### 2.1 Experimental approach to the problem

The BFR method was used to increase the power level and jump height of the CMJ and SJ to determine the occurrence of the PAPE effect in ice hockey players. The study was a controlled trial in which the participants (*N* = 20) of the experimental group constituted an experimental group for themselves. The same group of hockey players performed the same exercise program and control measurements 24 h apart on the first day under standard conditions, and on the second day using BFR. The experimental protocol was conducted during pre-season training, approximately one month before the start of league matches. The training carried out before the study, 12 days after the summer break, was introductory. In the period leading up to the experiment, all the subjects completed 10 training units on ice. Four of the units included elements of high-intensity effort in moderate volume.

Following the recommendations (23, 30, 34, 35) indicating that the PAPE effect is influenced by load parameters: number of repetitions, interval and form of restitution as well as type of movement performed, high-intensity ice skating repeated in ∼15-second sequences was selected as the exercise form. The control form of power recommended by Handford et al., Ciocca et al., and Vargas-Molina et al. (36–38) was the vertical jump CMJ and SJ result (PAPE effect).

The experimental design did not randomize the order of conditions. All participants completed the standard PAPE condition before the PAPE with BFR condition. This could have introduced an order effect on the results. However, it was minimized because the second condition was tested the following day, which did not limit the causal interpretation of differences between conditions.

### 2.2 Subjects

Twenty male professional ice hockey players (age: 17 ± 1 years; body mass: 76.1 ± 6.76 kg; body height: 181 ± 6 cm; BMI: 23.2 ± 2.1; experience in ice hockey training: 11 ± 2 years) from the Sports School of the National Ice Hockey Federation participated in this study. All subjects (in the case of 18 + years) or their parents or legal guardians (in the case of <18 years) provided their written consent to participate in this study after being informed of all procedures and risks involved in this study. They were in good health and reported no injuries and infections at the time of the study. The study was conducted by the Declaration of Helsinki and approved by the ethical committee of Jan Dlugosz University in Czestochowa (code: KE-U/2/2021). Power analysis was conducted using G*Power version 3.1.9.7 statistical software (Heinrich Heine University, Düsseldorf, Germany) to determine the required sample size. A repeated-measures analysis of variance (ANOVA) within factors (2 conditions and 6 time points) indicated that a sample size of 20 participants was required, assuming an α error of 0.05 and a statistical power of 80% to detect a small overall effect size (ES) of 0.24 (39).

### 2.3 Procedures

The experiment was divided into two sessions. The subjects were instructed to refrain from intense activity or significant physical effort for 24 h before the experiment. The experimental sessions were held 24 h apart to avoid fatigue and training effects (no training sessions were conducted between the first and second series of studies).

Before each session, the subjects performed a standardized off-ice warm-up focused on warming up the muscular system and stretching, and a 15 min on-ice warm-up with elements included in the specific effort during the experiment. The subjects performed the same exercise and control procedures in the first and second experimental series. In the second series, compression was applied to both limbs using Hytro Shorts (Hytro Limited, London, UK) for 3 min before and during the specific effort on the ice.

The 15-second effort duration was selected based on three components. First, the sport-specific demands of ice hockey shifts, which typically last 30–80 s, with high-intensity periods averaging 10–20 s (33, 40). Second, the 15 s interval replicates the average sprint duration during zone changes, the time required for defensive transitions and the corresponding energy system demands (predominantly ATP-PCr and glycolytic). Third, the chosen interval optimizes the PAPE window (41) and matches the time required to complete 2–3 game-action sequences.

**Figure 1:**
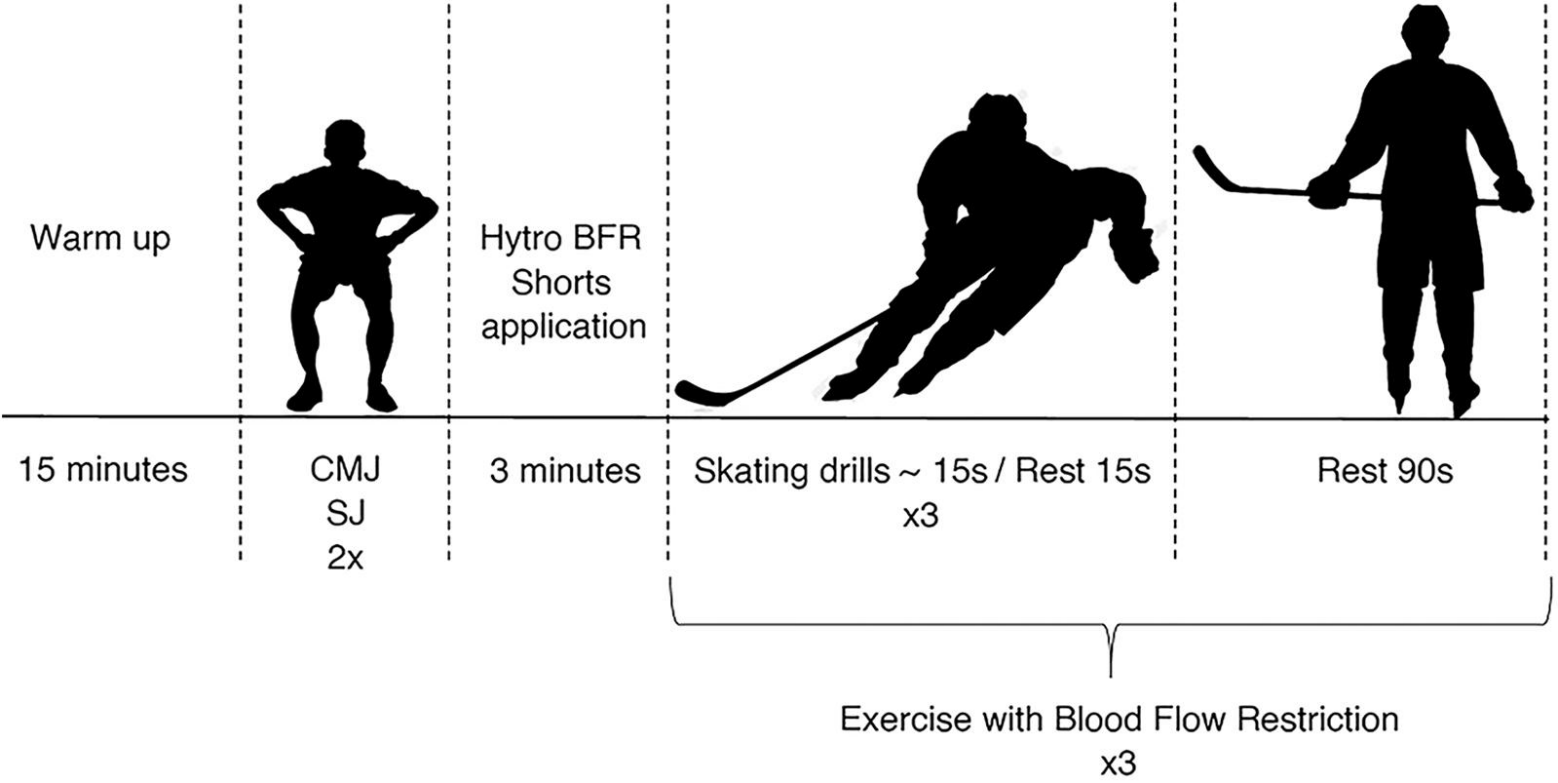
The PAPE exercise program using Hytro BFR shorts.

**Figure 2:**
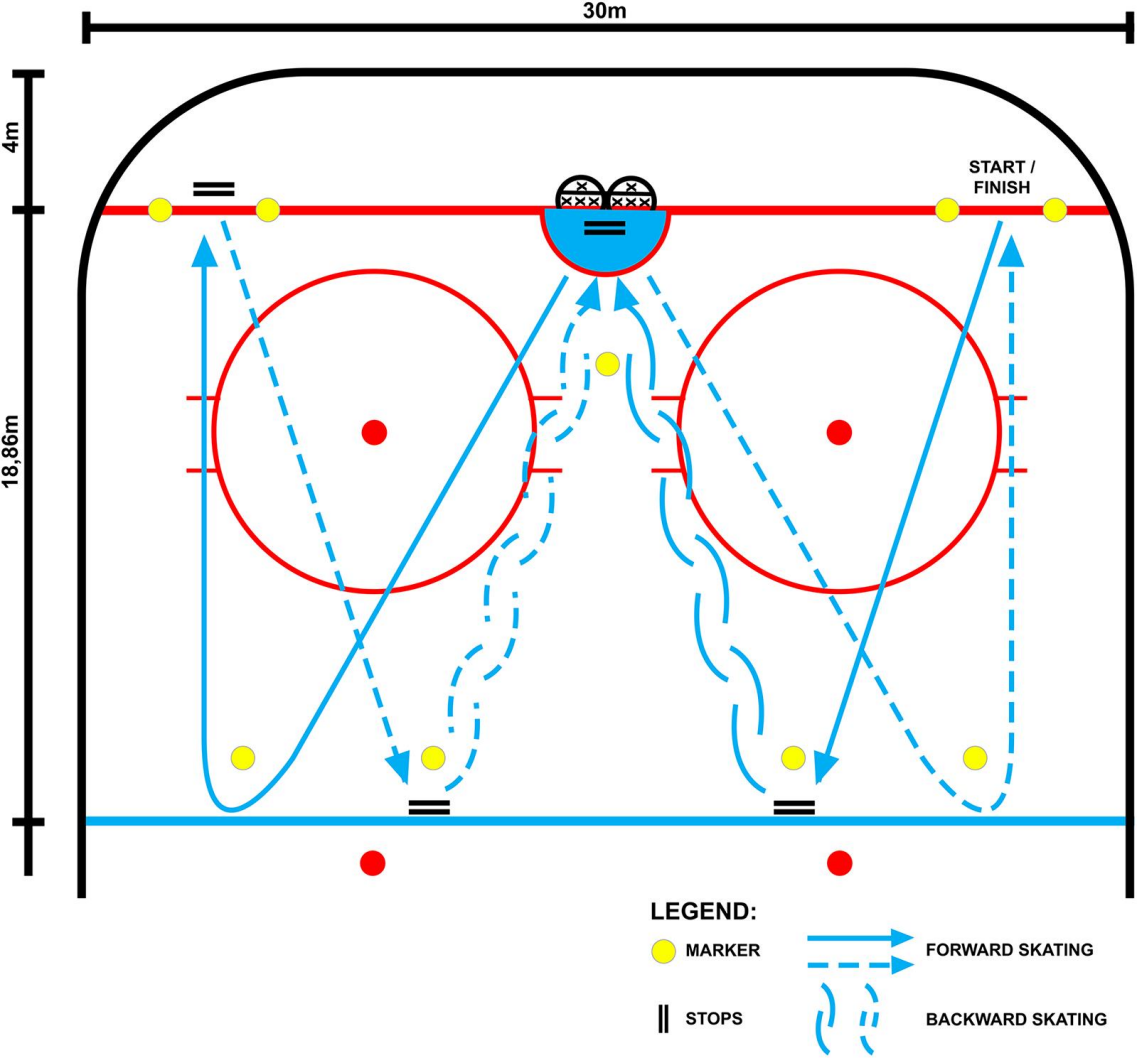
On-Ice protocol of the experiment exercise with and without occlusion.

The experimental procedure consisted of 6 elements, the structure of which is presented in Figure 1 and Table 1. The elements of the structure were: a standard 15 min warm-up on the ice; a 3 min break during which in the second experimental series the athlete was subjected to BFR by tightening the bands on Hytro Shorts; a vertical jump (CMJ and SJ) performed before the start of the effort on the ice; a specific effort performed on the ice consisting of skating forward, full stops, skating backwards and tight forward turns (Figure 2). The test protocol is based on the validated on-ice Repeated Ability Test (RAT), a comprehensive assessment of hockey performance that includes both offensive (forward skating/change of direction) and defensive (backward skating) movement patterns. The test replicates the sport-specific demands of ice hockey (42). The time of the effort∼15 s with a 15 s break performed 3 times in 3 series with a 90 s break; a 3 min break during which after the end of the effort the subjects in the second experimental series were deprived of the impact of BFR; a vertical jump (CMJ and SJ) in the 3rd, 6th, 9th, 12th and 15th minute after the end of the effort according to the procedure used for jumps before the start of the specific effort on the ice.

**Table 1:** Study design overview.

Stage	Day 1 STD	Day 2 BFR
Off-ice warm up	Yes	Yes
On-ice warm up—15 min	Yes	Yes
Baseline CMJ and SJ	Yes	Yes
BFR Shorts application	No	Yes (3 min before and during exercise)
PAPE on-ice exercise program	3 sets x 3 reps x 15 s work/15 s rest (90 s rest between sets)	3 sets x 3 reps x 15 s work/15 s rest (90 s rest between sets) (BFR during exercise)
BFR Shorts deapplication	No	Yes
Post-test CMJ and SJ	3rd, 6th, 9th, 12th, 15th minute	3rd, 6th, 9th, 12th, 15th minute

STD, standard conditioning; BFR, blood flow restriction conditioning; PAPE, post-activation performance enhancement; CMJ, countermovement jump; SJ, squad jump.

### 2.4 Power and height jump measurements

Lower limb peak power (Pp) and jump height (JH) were determined in vertical jump (CMJ and SJ). This control test measures the power of the lower limbs from a squat at a 90° angle at the knee joint. The subject performed a jump according to the protocol of Bosco et al. (43). In each attempt, the subject was instructed to perform a vertical jump with as much effort as possible to achieve the highest jump height (44). Based on the flight time (FT) measured using the Optojump measuring device (Microgate, Srl., Bolzano, Italy), Pp and JH were estimated. The power of the jumps was calculated from vertical jump height according to Sayers's unified formula (45):

Pp=(60.7×JH[cm])+(45.3×BM[kg])−2055

Pp is peak power [W], BM is the body mass [kg], JH is the vertical jump height [cm], 60.7; 45.3 and 2055 are constant coefficients.

### 2.5 On-ice performance assessments

In order to induce the PAPE effect, the subjects performed a specific effort on ice. The effort consisted of forward skating, backward skating in a ratio of 3:1, full stops, and tight forward turns (Figure 2).

### 2.6 Blood flow restriction (BFR)

The BFR effect was achieved using Hytro Shorts (Hytro Limited, London, UK) with built-in cuffs for the lower limbs. The size of Hytro Shorts was individually selected based on the thigh circumference and weight of the subject. Compression was achieved by tightening a 5 cm wide elastane cuff at level 2 at the most proximal part of each thigh, creating a compression band. Elastane cuffs were attached for 3 min before starting, and the compression level was maintained during the specific effort on the ice. After completing the effort on the ice, the pressure in the elastane cuff was released. The cuff is secured with a Velcro mechanism (YKK Fastening Corp, Tokyo, Japan and provides a standardized compression stimulus. In the experiment, the pressure used was at level 2, which according to previous studies (28) means that the applied pressure limiting the blood flow was at the level of 178 ± 13 mmHg, which causes 71 ± 5% of the limb occlusion pressure (28) and reported this way of method as a reliable for identifying sub occlusive pressures (29).

### 2.7 Statistical methods

All the statistical analyses were performed with OriginPro 2024 (OriginLab Corporation, USA). Mean and standard deviation were used to represent the average and the typical spread of values of all the measured variables. Statistical significance was set at α ≤ 0.05. The normality of the data distribution was verified using the Shapiro–Wilk test. The acute effects of the BFR on the dependent variables were examined by Two-Way Repeated Measures ANOVA (2 conditions and 6 time points). Analysis of variance was used with a Tukey *post-hoc* Test to determine whether and where differences existed in all measured variables between the conditions and each time point. If data were not normally distributed, pre-post differences were analyzed using the Paired Sample Wilcoxon Signed-Rank Test. The intervention's effect size (ES) was calculated using Cohen's guidelines. Threshold values for ES were >0.2 (small), >0.6 (moderate), >1.2 (large), and >2.0 (very large). The 95% confidence interval (CI) of Cohen's d was calculated.

## 3 Results

### 3.1 On-ice skating time

The Two-Way ANOVA (2 conditions and 3 time points) showed a significant effect for both conditions (*p* < 0.0001) and each set (*p* = 0.033), but no significant interaction effect (*p* = 0.127) for the on-ice skating performance. The Tukey *post-hoc* Test confirmed significant pairwise differences between conditions across all skating sets (*p* < 0.0001).

**Figure 3:**
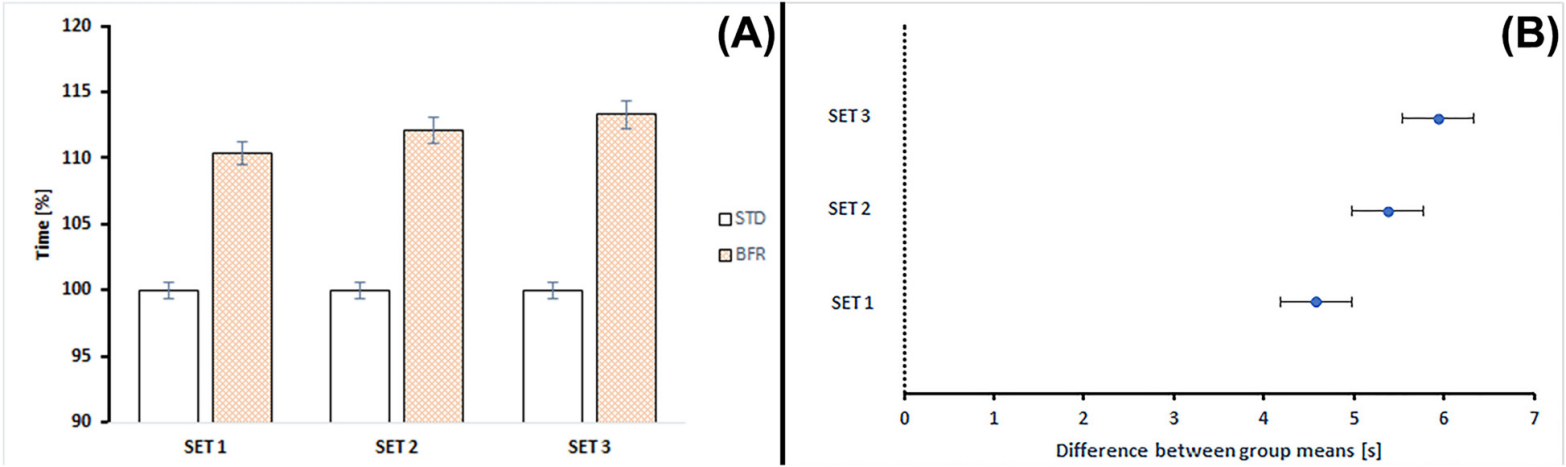
**(A)** Changes in time across on-ice skating sets, STD, standard conditioning, BFR, blood flow restriction conditioning. **(B)** Differences between the means of time on-ice skating results according to *post-hoc* analyses. The error bars are the standard error of the mean.

Furthermore, the on-ice skating times were significantly longer during BFR conditioning (from 4.57 to 5.93 of mean difference) as well as for the total time across all on-ice skating sets (15.89 of mean difference). The ES significantly affects each set and total time (Table 2 and Figures 3A,B).

**Table 2:** Comparison of differences on-ice skating in conditions after each set and total time.

Set	STD	BFR	Mdiff [%]	*p*-Value	ES		CI 95%
	M ± SD						
∑S1	44.28 ± 2.69	48.86 ± 3.79	10.33	<0.0001*	1.394	Large	0.416–2.371
∑S2	44.45 ± 2.65	49.83 ± 4.27	12.09	<0.0001*	1.514	Large	0.520–2.508
∑S3	44.70 ± 2.68	50.64 ± 4.59	13.28	<0.0001*	1.580	Large	0.576–2.585
∑S	133.44 ± 7.86	149.34 ± 11.98	11.91	<0.0001*	1.578	Large	0.574–2.581

STD, standard conditioning; BFR, blood flow restriction conditioning; M, mean; SD, standard deviation; Mdiff, mean difference; ES, effect size; CI, confidence interval; ∑S#, sum of time exercise in each Set#; ∑S, total exercise time from all sets.

* Indicates a statistically significant difference.

Figure 3A illustrates the percentage changes in skating time and the comparison between STD and BFR conditioning across time points. BFR conditioning showed longer time ranging from 10.33% to 13.28% compared to STD conditioning.

### 3.2 Jump height

The Two-Way ANOVA (2 conditions and 6 time points) did not show a significant effect of conditions (*p* = 0.150 for CMJ and *p* = 0.294 for SJ) and time points (*p* = 0.194 for CMJ and *p* = 0.056 for SJ), nor interaction of variables (*p* = 0.720 for CMJ and *p* = 0.526 for SJ) for JH.

The analysis of vertical jump performance demonstrated a significant difference between conditions for CMJ at the 1st time point measurement (*p* = 0.030), with the BFR conditioning achieving higher jump height (38.48 ± 6.28 cm) compared to the STD conditioning (36.75 ± 4.99 cm). Although statistically significant, this effect size was small (ES = 0.305).

For SJ performance, no statistically significant differences between conditions were observed at any time point (*p* = 0.089–0.750). Effect sizes were predominantly trivial (0.041–0.197), except for the baseline and 1st time point after intervention measurement, where small effects size were observed (0.522–0.562) (Table 3).

**Table 3:** Comparison of pre-post differences in jump height, conditions and each time points.

Vertical jump	Time point	STD	BFR	Mdiff [%]	*p*-Value	ES		CI 95%
	M ± SD							
CMJ [cm]	B	38.07 ± 4.97	38.17 ± 4.43	0.26	0.898	0.021	Trivial	0.855–0.898
	1	36.75 ± 4.99	38.48 ± 6.28	4.71	0.030*	0.305	Small	0.577–1.187
	2	36.47 ± 4.82	37.73 ± 5.84	3.46	0.112	0.235	Small	0.644–1.115
	3	37.13 ± 4.75	37.61 ± 6.64	1.30	0.541	0.083	Trivial	0.794–0.960
	4	36.75 ± 4.88	37.94 ± 6.56	3.23	0.134	0.206	Small	0.673–1.085
	5	36.76 ± 4.81	37.72 ± 5.59	2.62	0.224	0.184	Trivial	0.694–1.062
SJ [cm]	B	37.96 ± 4.94	37.57 ± 4.85	−1.01	0.605	0.522	Small	0.957–0.797
	1	36.64 ± 5.08	37.91 ± 6.29	3.47	0.089	0.562	Small	0.657–1.101
	2	36.28 ± 5.12	37.03 ± 6.79	2.07	0.313	0.125	Trivial	0.753–1.002
	3	36.28 ± 5.41	36.52 ± 6.36	0.65	0.750	0.041	Trivial	0.750–1.004
	4	35.54 ± 5.37	36.66 ± 6.01	3.14	0.135	0.197	Trivial	0.682–1.075
	5	36.22 ± 5.92	36.97 ± 5.81	2.06	0.315	0.128	Trivial	0.750–1.005

STD, standard conditioning; BFR, blood flow restriction conditioning; B, baseline; M, mean; SD, standard deviation; Mdiff, mean diference; ES, effect size; CI, confidence interval.

* Indicates a statistically significant difference.

**Figure 4:**
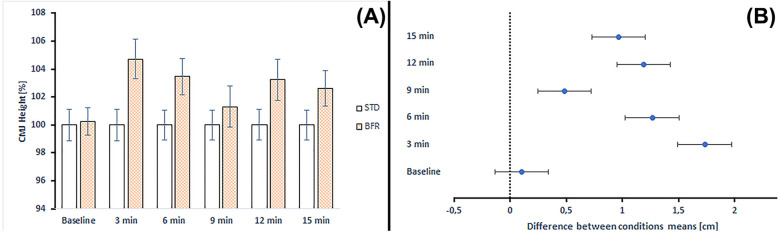
**(A)** Changes in CMJ height and comparison between standard and BFR conditioning across time points. STD, standard conditioning; BFR, blood flow restriction conditioning. **(B)** Differences between mean CMJ height values at each time point according to *post-hoc* analyses. Error bars represent the standard error of the mean.

Figure 4A illustrates the percentage changes in CMJ height and the comparison between STD and BFR conditioning across time points. BFR conditioning demonstrated improvements ranging from 0.26% to 4.71% compared to STD conditioning.

**Figure 5:**
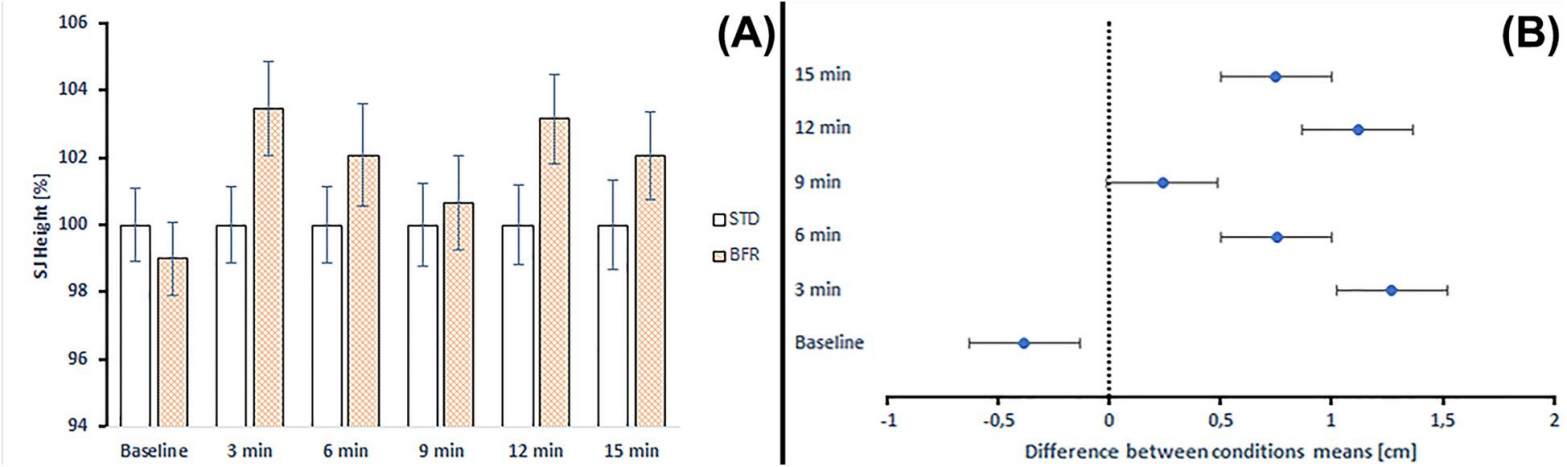
Changes in SJ height and comparison between standard and BFR conditioning across time points. STD, standard conditioning; BFR, blood flow restriction conditioning. **(B)** Differences between mean CMJ height values at each time point according to *post-hoc* analyses. Error bars represent the standard error of the mean.

Figure 5A illustrates the percentage changes in SJ height and the comparison between STD and BFR conditioning across time points. BFR conditioning demonstrated improvements ranging from 0.65% to 3.47% compared to STD conditioning. The only exception was observed at baseline, where BFR results were −1.01%.

Moreover, the absolute JH values were higher after BFR conditioning compared to the corresponding values in CMJ (from 0.10 to 1.73 of mean difference) and SJ (from 0.23 to 1.27 of mean difference). The only exception was a lower absolute JH value at baseline in SJ following BFR conditioning (mean difference = 0.384) (Figures 4B, 5B).

Analysis of pre–post changes compared to baseline indicated that in the STD conditioning, CMJ height decreased at all post-intervention time points (from −4.20% to −2.46%), with no statistically significant decrease (*p* = 0.333–0.839). In the BFR conditioning, changes ranged from −1.45% to −0.61%, with all comparisons to baseline being non-significant (*p* = 0.980–0.999).

Pre–post changes compared to baseline indicated that in the STD conditioning, SJ height decreased at all post-intervention time points (from −6.36% to −3.47%), with statistically significant decrease observed at 4th time point (*p* = 0.019). In the BFR conditioning, changes ranged from −2.80% to −1.44%, with all comparisons to baseline being non-significant (*p* = 0.980–0.999) (Table 4).

**Table 4:** Comparison of pre-post differences in jump height, conditions and each time points with baseline.

Vertical jump	Time point	STD	*p*-Value	BFR	*p*-Value
		Mean difference [%]		Mean difference [%]	
CMJ [cm]	1:B	−3.46	0.551	0.81	0.998
	2:B	−4.20	0.333	−1.14	0.993
	3:B	−2.46	0.839	−1.45	0.980
	4:B	−3.47	0.547	−0.61	0.999
	5:B	−3.45	0.556	−1.18	0.992
SJ [cm]	1:B	−3.47	0.484	0.89	0.997
	2:B	−4.42	0.219	−1.44	0.977
	3:B	−4.41	0.222	−2.80	0.715
	4:B	−6.36	0.019*	−2.42	0.822
	5:B	−4.57	0.189	−1.60	0.964

STD, standard conditioning; BFR, blood flow restriction conditioning; B, baseline.

* Indicates a statistically significant difference.

### 3.3 Jump power

The Two-Way ANOVA (2 conditions and 6 time points) did not show a significant effect of conditions (*p* = 0.141 for CMJ and *p* = 0.273 for SJ) and time points (*p* = 0.182 for CMJ and *p* = 0.062 for SJ), nor interaction of variables (*p* = 0.694 for CMJ and *p* = 0.485) for peak power.

The analysis of peak power revealed a significant difference between conditions for CMJ at the 1st time point measurement (*p* = 0.023), with the BFR conditioning achieving higher peak power (49.13 ± 5.19 W/kgBM) compared to the STD conditioning (47.65 ± 4.15 W/kgBM). Although statistically significant, this effect size was small (ES = 0.315).

For SJ peak power, no statistically significant differences between conditions were observed at any time point (*p* = 0.067–0.652). Effect sizes were predominantly trivial (0.057–0.234), except for the 1st time point measurement, where small effects were observed (0.234) (Table 5).

**Table 5:** Comparison of pre-post differences in jump power, conditions and time points.

Vertical jump	Time point	STD	BFR	Mdiff [%]	*p*-Value	ES		CI 95%
		M ± SD						
CMJ [W/kgBM]	B	48.76 ± 4.02	48.84 ± 3.61	0.15	0.906	0.021	Trivial	0.856–0.898
	1	47.65 ± 4.15	49.13 ± 5.19	3.10	0.023*	0.315	Small	0.567–1.197
	2	47.47 ± 3.99	48.51 ± 4.76	2.19	0.107	0.237	Small	0.643–1.116
	3	48.01 ± 3.89	48.46 ± 5.44	0.94	0.482	0.095	Trivial	0.782–0.972
	4	47.66 ± 3.89	48.69 ± 5.38	2.17	0.109	0.219	Small	0.660–1.099
	5	47.69 ± 3.88	48.51 ± 4.64	1.71	0.205	0.192	Trivial	0.687–1.070
SJ [W/kgBM]	B	48.69 ± 3.98	48.32 ± 3.89	−0.74	0.544	0.094	Trivial	0.971–0.783
	1	47.60 ± 4.20	48.70 ± 5.14	2.30	0.067	0.234	Small	0.645–1.114
	2	47.30 ± 4.21	48 ± 5.43	1.48	0.238	0.144	Trivial	0.734–1.022
	3	47.30 ± 4.38	47.57 ± 5.11	0.56	0.652	0.057	Trivial	0.820–0.933
	4	46.76 ± 4.29	47.65 ± 4.94	1.89	0.138	0.192	Trivial	0.686–1.071
	5	47.31 ± 4.77	47.93 ± 4.69	1.31	0.297	0.131	Trivial	0.746–1.009

STD, standard conditioning; BFR, blood flow restriction conditioning; B, baseline; M, mean; SD, standard deviation; Mdiff, mean difference; ES, effect size; CI, confidence interval; BM, body mass.

* Indicates a statistically significant difference.

**Figure 6:**
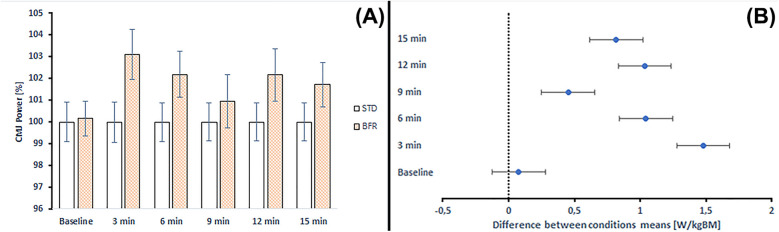
**(A)** Changes in CMJ peak power and comparison between standard and BFR conditioning across time points. STD, standard conditioning; BFR, blood flow restriction conditioning. **(B)** Differences between mean CMJ peak power values at each time point according to *post-hoc* analyses. Error bars represent the standard error of the mean.

Figure 6A illustrates the percentage changes in CMJ peak power and the comparison between STD and BFR conditioning across time points. BFR conditioning revealed improvements ranging from 0.15% to 3.10% compared to STD conditioning.

**Figure 7:**
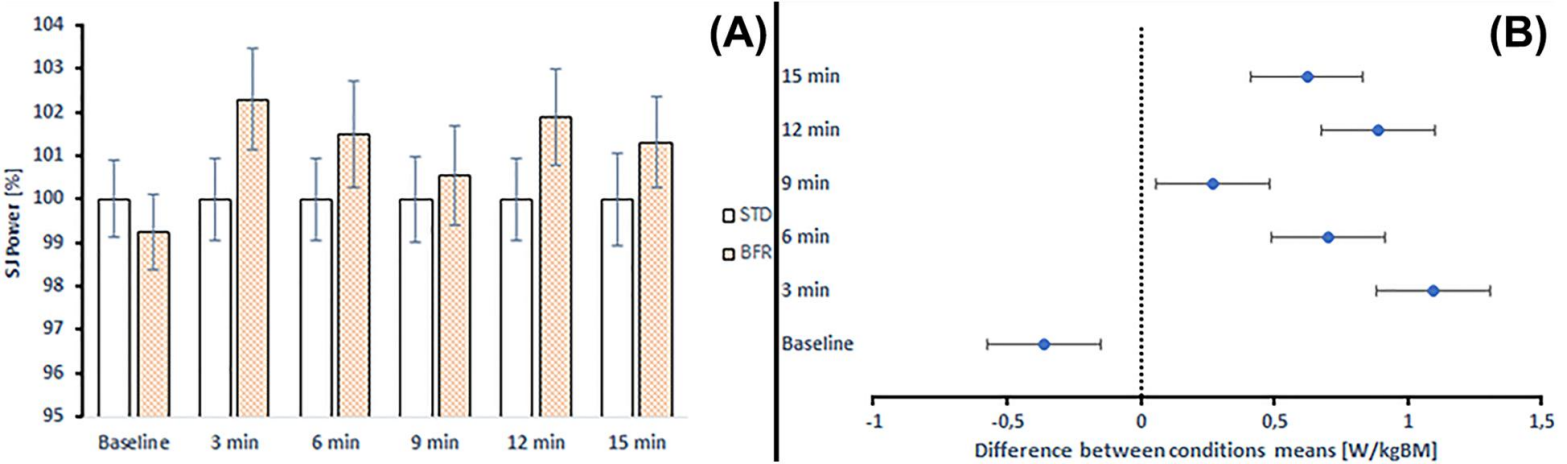
**(A)** Changes in SJ peak power and comparison between standard and BFR conditioning across time points. STD, standard conditioning; BFR, blood flow restriction conditioning. **(B)** Differences between mean SJ peak power values at each time point according to *post-hoc* analyses. Error bars represent the standard error of the mean.

Figure 7A illustrates the percentage changes in SJ peak power and the comparison between STD and BFR conditioning across time points. BFR conditioning revealed improvements ranging from 0.56% to 2.30% compared to STD conditioning. The only exception was observed at baseline, where BFR results were −0.74%.

Moreover, the peak power values were higher after BFR conditioning compared to the corresponding values in CMJ (from 0.07 to 1.47 of mean difference) and SJ (from 0.26 to 1.09 of mean difference). The only exception was a lower peak power value at baseline in SJ following BFR conditioning (mean difference = −0.360) (Figures 6B, 7B).

Analysis of pre–post changes compared to baseline indicated that in the STD conditioning, CMJ peak power decreased at all post-intervention time points (from −2.64% to −1.53%), with no statistically significant decrease (*p* = 0.341–0.850). In the BFR conditioning, changes ranged from −0.76% to −0.29%, with all comparisons to baseline being non-significant (*p* = 0.992–0.999).

Pre–post changes compared to baseline indicated that in the STD conditioning, SJ peak power decreased at all post-intervention time points (from −3.95% to −2.23%), with statistically significant decrease observed at 4th time point (*p* = 0.019). In the BFR conditioning, changes ranged from −1.56% to −0.66%, with all comparisons to baseline being non-significant (*p* = 0.795–0.988) (Table 6).

**Table 6:** Comparison of pre-post differences in jump power, conditions and each time points with baseline.

Vertical jump	Time point	STD	*p*-Value	BFR	*p*-Value
		Mean difference [%]		Mean difference [%]	
CMJ [W/kgBM]	1:B	−2.28	0.509	0.59	0.997
	2:B	−2.64	0.341	−0.66	0.995
	3:B	−1.53	0.850	−0.76	0.992
	4:B	−2.26	0.520	−0.29	0.999
	5:B	−2.19	0.554	−0.67	0.995
SJ [W/kgBM]	1:B	−2.23	0.450	0.76	0.988
	2:B	−2.84	0.190	−0.66	0.994
	3:B	−2.84	0.189	−1.56	0.795
	4:B	−3.95	0.019*	−1.40	0.862
	5:B	−2.82	0.195	−0.81	0.985

STD, standard conditioning; BFR, blood flow restriction conditioning; B, baseline; M, mean; SD, standard deviation; ES, effect size; CI, confidence interval; BM, body mass.

* Indicates a statistically significant difference.

## 4 Discussion

The experiment aimed to analyze the PAPE effect and intensified BFR in ice hockey players towards the power increase in the lower limb power expressed in the height of the CMJ and SJ. According to our knowledge, this is the first research combining the PAPE effect with BFR using a specific on-ice exercise for hockey players. The intensified BFR was supposed to stimulate a higher level of lower limb power expressed in the increase in the height obtained in CMJ and SJ.

Previous studies have shown the effectiveness of loads causing PAPE effect classified as low or moderate intensity (46–48). In the training practice of team games, except for volleyball, there is no such compilation of intensity in the game. During the game, a very rare situation occurs when low-intensity effort is preceded by a movement that requires high-power manifestation. Usually, game situations leading to obtaining an optimal position in ice hockey are preceded by high-intensity anaerobic work (49, 50). Therefore, training effectiveness requires identifying conditions that promote improvements in on-ice power among ice hockey players through specialized training with varied effort characteristics. For this purpose, our research used a specific effort reflecting in-game movements during attack and defense, which was combined with an effort requiring high power generation, such as the CMJ and SJ jump (simulating an acceleration over a distance of 1–2 steps). The research procedure was intended to determine the potential of using PAPE and enhancing PAPE by BFR in specific training conditions. Another goal was identifying the most advantageous time for performing speed exercises following anaerobic work.

The research results showed an extended in the range of 6% to 12% (individually in players) of the total time (sum of the time of three sets of exercises) intensity of specific work on ice in the conditions of BFR application. Given that this extension was similar for each set 1–3, magnitude from 5%–13%, 7%–12%, and 8%–13%, respectively, the impact of fatigue on the increased exercise time included in the PAPE program can be eliminated. A characteristic feature of the studied group of hockey players was the high diversity of players' reactions to the BFR application. Individualizing the compression ratio may reduce these differences (25). However, this approach requires further experimental investigation. Previous research has been based on PAPE triggering efforts in interaction with BFR at a significantly lower intensity than in our studies (51, 52). The results of this research indicate a limitation of BFR conditioning under constant work intensity. There was significant variation among the subjects in terms of BFR tolerance and readiness for anaerobic exercise.

Jump height after anaerobic on ice exercise without BFR factor showed a non-significant decrease in CMJ values ranging from 2.46%–4.20% and in SJ ranging from 3.47% to 6.36%. These results have not been confirmed by other studies on team sport athletes (53–55). The use of BFR as a supplement to effort within the PAPE program produced only non-significant effects on changes in jump height and peak power. Both indices maintained a downward trend, with decreases of 1.53%–2.64% and 0.29%–0.76% for CMJ, and 2.23%–3.95% and 0.66%–1.56% for SJ, respectively. The observed pattern of changes in the case of pre-exercise PAPE and in the combination of PAPE with BFR is consistent with the studies by Konrad et al. (56) and Prieske et al. (57).

Most studies on the effect of PAPE on jump height conducted in team sports players, such as volleyball (58–60), football (61), and handball (62), indicate a positive effect of effort preceding the generation of high power in a short time. However, fatigue or energy exhaustion during the exercises from the PAPE program has been identified as a limiting factor (63). The lack of a significant improvements in jump height in this study may be attributed to the specific effort used. The exercise program, which was the aim of the study, reflected training conditions in which the effort was repeated many times and was of a strongly anaerobic nature. As highlighted by Tillin and Bishop (41), excessive exercise loads may induce fatigue, limiting the response to PAPE interventions (64, 65). Furthermore, individual variability in responses suggests the necessity of personalized adjustments in activation intensity and load volume to match the unique characteristics of athletes. This is particularly important given the observed trends in power indices, which paralleled those in jump height.

### 4.1 Limitations and future research

The conducted study has certain limitations, that other authors have pointed out in this type of intervention (66–70). The limitations in our study are related to the influence of the intensity of training intervention, muscle temperature and intracellular fluid content on the effect. Potential fatigue caused by repeated maximal jumping efforts could have masked subtle strengthening effects. In addition, the study used a constant arterial occlusion pressure across all participants, lack of individualization for vascular or muscular characteristics, may potentially limiting the effectiveness or comparability of the BFR intervention. Limitations include the small sample size of 20 participants.

Although highly specialized, it restricts the generalizability of findings to broader athletic populations with varying training backgrounds, experience level, sport disciplines and female athletes. Environmental factors, such as the low ambient temperature of the ice rink, could have influenced muscle performance and blood flow dynamics, adding complexity to the results' interpretation. These observations emphasize the need for future research with larger, more diverse cohorts, long-term performance tracking, and refined protocols to enhance the reliability and applicability of findings to athletic practice.

### 4.2 Study implications

Future research should consider examining individual BFR pressures, more extended recovery periods between assessments, and integration of direct physiological markers (e.g., EMG, muscle temperature, or blood flow restriction dynamics), which could also improve the understanding of underlying mechanisms.

Additionally, research comparing BFR with other traditional PAPE programs (e.g., plyometric or resistance-based protocols) in endurance sport may help improve practical applications.

## 5 Conclusions

Applying specific effort on ice, consisting of 9 repetitions of 15 s of high-intensity skating separated by a 90 s rest to induce the PAPE effect, is ineffective. There were no significant statistical differences in the level of change in lower limb power within 15 min after the end of exercise. The BFR application during specific effort and the 3 min preceding the effort resulted in a statistically significant reduction in work intensity. In the 15 min after completing the work, the reduction in lower limb power was lower than in the absence of BFR, but the changes were not statistically significant. In both cases of PAPE and PAPE with BFR, high individual variability, like the hockey players' reactions to the applied intervention, was demonstrated. However, attributing the improvement in lower limb power in some of the study participants solely to the PAPE or PAPE with BFR effect generated by the initial conditioning activity is not justified. The numerous physical and cognitive factors that influence final performance make it difficult to identify the impact of PAPE and BFR on each subject. For these reasons, the individualization of stimuli is essential. It should consider the individual athlete's profile regarding susceptibility to the PAPE and BFR program and the fatigue it may cause. The use of PAPE and BFR to achieve an ongoing power-enhancing effect under the specific conditions of ice hockey training has limited application, and the effect obtained is not unequivocal for the entire team.

## Data Availability

The raw data supporting the conclusions of this article will be made available by the authors, without undue reservation.
